# Pentoxifylline and Norcantharidin Modify p62 Expression in 2D and 3D Cultures of B16F1 Cells

**DOI:** 10.3390/ijms25105140

**Published:** 2024-05-09

**Authors:** José Luis González-Quiroz, Juan Moisés Ocampo-Godínez, Victoria Noemi Hernández-González, Ruth Angélica Lezama, Elba Reyes-Maldonado, Armando Vega-López, María Lilia Domínguez-López

**Affiliations:** 1 Laboratorio de Inmunoquímica I, Departamento de Inmunología, Escuela Nacional de Ciencias Biológicas, Instituto Politécnico Nacional, Prolongación de Carpio y Plan de Ayala S/N, Santo Tomás, Ciudad de Mexico 11340, Mexico; jose.luis.gon.q.91@gmail.com (J.L.G.-Q.);; 2 Laboratorio de Bioingeniería de Tejidos, Departamento de Estudios de Posgrado e Investigación, Universidad Nacional Autónoma de México, Ciudad de Mexico 04360, Mexico; 3 Laboratorio de Hematopatología, Departamento de Morfología, Escuela Nacional de Ciencias Biológicas, Instituto Politécnico Nacional, Ciudad de Mexico 11340, Mexico; 4 Laboratorio de Toxicología Ambiental, Departamento de Ingeniería en Sistemas Ambientales, Escuela Nacional de Ciencias Biológicas, Instituto Politécnico Nacional, Ciudad de Mexico 07738, Mexico

**Keywords:** melanoma, 3D cultures, autophagy, p62

## Abstract

Three-dimensional cell cultures have improved the evaluation of drugs for cancer therapy, due to their high similarity to solid tumors. In melanoma, autophagy appears to show a dual role depending on the progression of the disease. p62 protein has been proposed for the evaluation of autophagic flux since its expression is an indicator of the state of autophagy. Pentoxifylline (PTX) and Norcantharidin (NCTD) are drugs that have been shown to possess anticancer effects. In this work, we used B16F1 mouse melanoma cells in two-dimensional (2D) monolayer cultures and three-dimensional (3D) spheroids to test the effect of PTX and NCTD over the p62 expression. We analyzed the effect on p62 expression through Western blot and immunofluorescence assays. Our results indicate that PTX decreases p62 expression in both cell culture models, while Norcantharidin increases its expression in 3D cultures at 24 h. Therefore, these drugs could have a potential therapeutic use for the regulation of autophagy in melanoma, depending on the state of evolution of the disease.

## 1. Introduction

Melanoma is one of the most common types of cancer caused by the transformation of melanocytes. In advanced stages, it is considered an aggressive type of cancer that is difficult to treat due to its risk of metastasis [1]. To evaluate new chemotherapies against melanoma, human and mouse cell lines, such as the B16F1 mouse cell line, are often used [2]. These cancer cell lines are commonly used in two-dimensional (2D) monolayer cell culture models to test different drugs. However, 3D cultures have become relevant in the field of oncotherapy because they provide a better model to evaluate the effect of different drugs in the context of a tumor [3]. They confer similar properties to an in vivo 3D tumor environment with the expression of molecules that recapitulate tumor physiology [4,5,6].

Autophagy is an adaptive cellular process that maintains homeostasis under stress conditions and serves as a quality control mechanism for organelles and proteins. This mechanism can be triggered by various intra- or extracellular stimuli [7]. The initial step of autophagy is the formation of a double-membrane phagophore in the endoplasmic reticulum. Subsequently, primarily phagophore elongation is carried out by the class III PI3K complex. During elongation, the binding of several proteins to the autophagophore membrane occurs, including the recruitment of LC3 [8]. LC3 undergoes cleavage by ATG4, forming LC3BI, and is subsequently conjugated with phosphatidylethanolamine to form LC3BII. LC3BII is capable of binding to the autophagosome membrane and functions as an anchor protein for the selection of target proteins to be introduced into the autophagosome for degradation [9]. These proteins are selected by the SQSTM1 protein, also known as p62. Polyubiquitinated proteins bind to p62 and deliver them to the autophagosome through its interaction with the LIR domain of LC3BII [10,11]. In cancer, autophagy appears to have a dual role, as it is involved in both tumor suppression and tumor development, causing in some cases resistance to anticancer treatments [12].

PTX is a methylxanthine derivative with vasodilatory properties, which has been used to treat cardiac and cerebrovascular conditions [13]. However, PTX has recently been tested as a potential chemotherapy for several types of cancer [14]. Regarding this, PTX has been found to inhibit STAT3 phosphorylation in the JAK/STAT pathway in a murine melanoma model, decreasing angiogenesis and tumor metastasis [15,16]. Furthermore, it decreased proliferation and induced stress-dependent endoplasmic reticulum autophagy in human melanoma cell lines A375 and MeWo [17].

NCTD is the demethylated form of cantharidin obtained from the *Mylabris* beetle and has been used in traditional Chinese medicine, showing different antitumor effects [18]. In melanoma, NCTD induces apoptosis through the activation of caspases caused by overexpression of BAX and downregulation of Bcl-2 [19,20]. Furthermore, NCTD causes mitophagy-mediated apoptosis in several melanoma cell lines, by increasing LC3 expression and decreasing p62 [21]. In this work, we evaluated the effect of PTX and NCTD on autophagy in B16F1 cells using 2D and 3D cell culture models.

## 2. Results

### 2.1. Two-Dimensional and Three-Dimensional Cell Cultures

**Figure 1:**
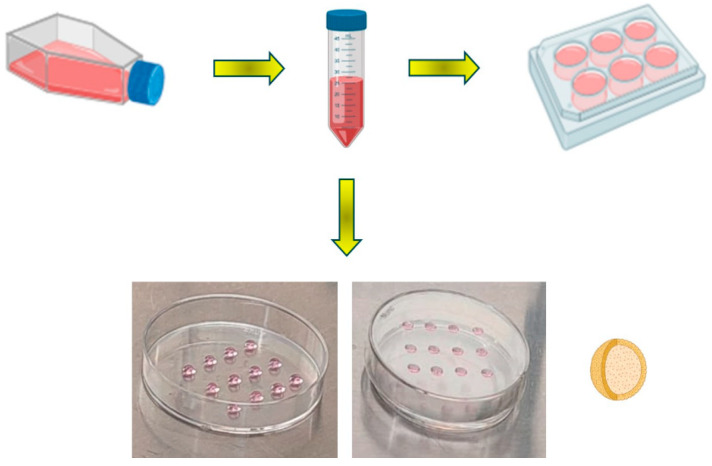
Schematic representation of the methodology to perform 2D and 3D cultures of mouse B16F1 melanoma cells. Two-dimensional cultures are obtained using the traditional protocol in sterile culture dishes. Three-dimensional cultures are constructed through the hanging drop technique in sterile Petri dishes.

The traditional protocol was followed to obtain cultures in 2D plates. Three-dimensional cultures were obtained using the hanging drop technique. This technique consists of using a Petri dish, where cells in suspension (due to gravity) fall to the bottom of the drop, inducing the formation of spheroids [22]. Thus, within the drop, cellular interactions and the conditions of the surrounding environment favor the formation of spheroids by aggregation. This model allowed us to replicate in vitro similar conditions of melanoma tumors (Figure 1). This method proved to be very efficient and low-cost compared to other methods used to construct spheroids [5,23].

### 2.2. Treatment of 2D and 3D Cell Culture Models with PTX and NCTD

**Figure 2:**
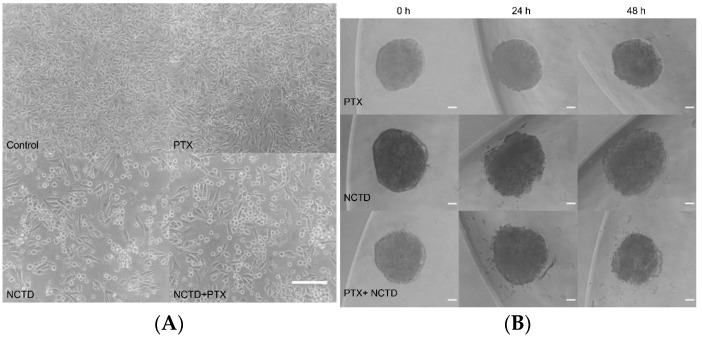
Effects of PTX and NCTD on 2D and 3D cell culture models of B16F1 cells. (**A**) In 2D cultures, B16F1 cells of the control group show their characteristic morphology of elongated fibroblastoid cells. Likewise, PTX-treated cells do not show visible changes in their morphology. However, NCTD and PTX+NCTD-treated cells show changes in their shape and size. Scale bar 50 μm. (**B**) Spheroids treated with NCTD and PTX+NCTD cause lower density and detachment of some cells from the proliferation zone, forming satellites at 24 and 48 h. Scale bar 10 μm.

Treatments with NCTD and the combination of both PTX+NCTD induced evident morphological changes in B16F1 cells. In the 2D cultures, NCTD and PTX+NCTD treated cells presented a reduction in their cytoplasm, showing a more compact and rounded shape compared to untreated controls (Figure 2A). In spheroids, the NCTD and PTX+NCTD groups showed a lower cell density in the proliferation zone. Some of the cells in this area are detached in the manner of satellites, being compatible with the phenomenon of spheroid lysis (Figure 2B).

### 2.3. Autophagy p62 Expression in 2D and 3D Cultures

**Figure 3:**
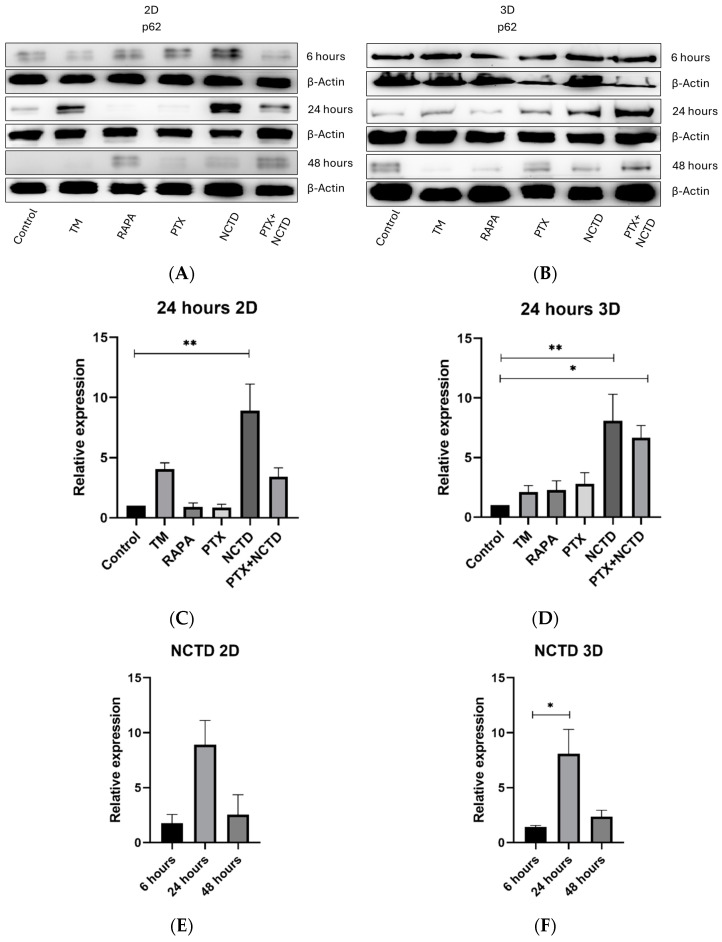
Differential expression of p62 in 2D and 3D culture models by Western blot analysis. (**A**) Representative image of p62 expression in 2D cell cultures. (**B**) Representative image of p62 expression in 3D cultures. (**C**) There is a significant increase only in the NCTD group at 24 h compared with the control group. (**D**) Three-dimensional cultures show a significant increase in p62 expression in the NCTD and PTX+NCTD groups at 24 h compared with the control group. (**E**,**F**) Both 2D and 3D models show a high expression of p62 at 24 h in the NCTD group, being only significant in the 3D model. Analyzes were performed in three independent experiments. The relative expression was normalized based on β-actin expression. The graphs represent the quantitative values of the intensity of the bands of figures (**A**,**B**). *p* < 0.05 = * and *p* < 0.01 = **.

Since rapamycin induces autophagy and tunicamycin induces endoplasmic reticulum stress, we used them as positive controls to evaluate p62 expression. We observed that tunicamycin increased p62 expression at 24 h with a subsequent decrease at 48 h (Figure 3A). We found that p62 expression decreased in 2D cell cultures treated with PTX at 24 and 48 h, while NCTD increased p62 expression at 6 and 24 h (Figure 3A). Regarding the 3D model, we found that PTX-treated spheroids decreased p62 expression at 24 h and 48 h (Figure 3B). NCTD-treated spheroids exhibited higher p62 expression at 24 h, but decreased at 48 h (Figure 3B,C). For both 2D and 3D cultures, the combination of PTX+NCTD significantly increased p62 expression at 24 h of treatment (Figure 3B–D). A relative expression analysis of p62 was performed in 2D and 3D cultures at the time points tested, finding a similar pattern in which p62 was highly expressed at 24 h but decreased at 48 h (Figure 3D–F).

### 2.4. Expression of p62 in 2D and 3D Cultures by Immunofluorescence

**Figure 4:**
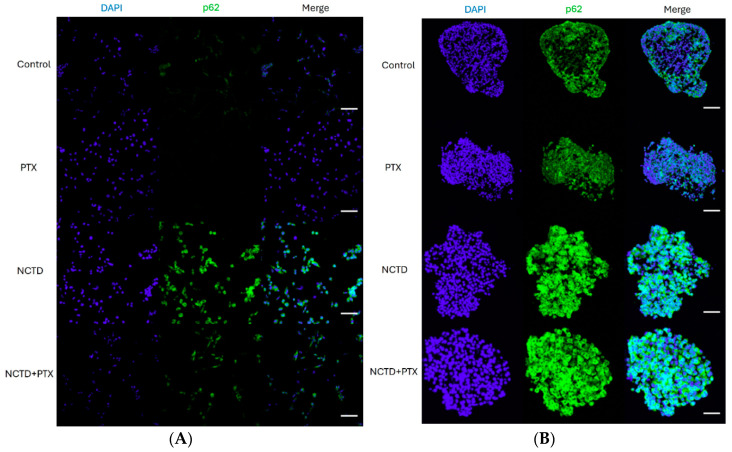
Expression of p62 in 2D and 3D cell culture models after 24 h of treatment by immunofluorescence. (**A**) Cells from untreated 2D cultures show a slight basal expression of p62 that is no longer observed in the PTX group. NCTD highly increases p62 expression and is related to cellular morphological changes. Cells treated with PTX+NCTD also show clear expression of p62 and exhibit morphological changes, but they are not superior to those in the group treated with NCTD alone. (**B**) Sections of untreated spheroids show basal expression of p62. Control and PTX-treated spheroids show labeling in isolated areas along the spheroid, while NCTD and PTX+NCTD spheroids show labeling in almost all cells of the spheroid. Cell nuclei in blue are stained with DAPI for both cultures. Scale bar 20 μm.

The effect on p62 expression of PTX, NCTD, and the combination of both drugs was analyzed by immunofluorescence in 2D and 3D cultures at 24 h. In 2D cultures, untreated controls showed basal expression of p62, which decreased when cells were treated with PTX. NCTD induced high expression of p62. Interestingly, although cells treated with PTX+NCTD also showed high expression of p62, it was not higher than cells treated with NCTD alone (Figure 4A). For 3D cultures, basal expression of p62 was observed in some areas of control and PTX spheroids at 24 h. However, spheroids treated with NCTD and NCTD + PTX showed strong expression of p62 in all spheroid cells (Figure 4B).

## 3. Discussion

Recently, 3D cell cultures have become important for anticancer drug screening, offering great similarity to solid tumors in vitro [3,5]. Spheroids provide a niche with several layers of cells that allow a better evaluation of different markers under different treatment models. This reduces the use of animals for the screening of new therapies [24]. Here, we constructed 3D spheroids of mouse B16F1 melanoma cells using the hanging drop method to test the effect of PTX and NCTD. We obtained spheroids with well-defined cell proliferation and a senescent zone. This 3D model allowed us to demonstrate part of the effect of NCTD and PTX. In this work, we demonstrate that NCTD promotes high expression of p62 which in turn is related to important morphological changes in melanocytes. The p62 protein is a scaffold molecule with multiple domains. This molecule is also known as sequestosome 1 (p62//SQSTM 1) and can interact with phagosomes through interaction with LC3. Because p62 possesses multifunctional and signaling properties involved in the regulation of autophagy and apoptosis, it is believed to play an important role in cancer [25,26,27,28]. Its regulation has been the subject of several studies, and as a result, several modulators of autophagy have gained importance in melanoma [29]. In the early stages of the disease, autophagy promotes the removal of misfolded or mutated proteins [30]. This alteration in autophagy, together with the accumulation of p62, contains tumor progression [31]. Once metastasis occurs, tumor cells can activate autophagy and p62 levels decrease. This reactivation of autophagy, under treatments such as chemotherapy, makes tumor cells resistant, preventing their death [32]. Interestingly, we found that NCTD induces high expression of p62 in both 2D and 3D models at 24 h. Indeed, in our 3D model, NCTD promoted spheroid disaggregation. This is relevant since higher autophagic activity has been reported in 3D cultures, which usually leads to lower effectiveness of anticancer drugs [33,34]. Despite the antitumoral properties of NCTD, some studies have shown that p62 accumulation is a marker of poor prognosis in different types of cancer [35,36,37]. However, in melanoma, it has been shown that the decrease in p62 in the late stages of the disease is related to metastasis [38]. Therefore, since autophagy in melanoma improves tumor survival, the use of an autophagy inhibitor such as NCTD could be useful in the advanced stages of the disease to contain metastasis.

On the other hand, PTX also possesses anticancer effects [14,39]. Previous studies have shown that it has antimetastatic properties in human and mouse melanoma cell lines [40,41]. In this work, we demonstrate that PTX can increase autophagy in mouse B16F1 melanoma cells through the degradation of p62 in 2D and 3D cultures, promoting the preservation of cell morphology and avoiding spheroids lysis (Figure 3A,B). This effect could be a result of increased endoplasmic reticulum stress triggered by PTX, which is a mechanism that degrades misfolded proteins and can activate autophagy in cells [17].

In conclusion, NCTD is a potent inducer of p62, causing important morphological changes in B16F1 melanoma cells and spheroid lysis in vitro. Future in vivo studies are necessary to determine the potential role of NCTD in controlling metastasis in advanced stages of the disease.

## 4. Materials and Methods

### 4.1. Two-Dimensional (2D) and Three-Dimensional (3D) Cell Culture Models

For cell culture, murine melanoma cell line B16F1 obtained from the American Type Culture Collection (ATCC, #CRL, 1872, Bethesda, MD, USA) was used. It was cultured using DMEM/F12 medium + GlutaMAX^TM^ (cat# 10565-018, Gibco, Billings, MT, USA) supplemented with 10% fetal bovine serum at 37 °C in 5% CO_2_. Cells were detached with Trypsin-EDTA (cat# 25200-056, Gibco) when they reached >80% of confluence. Then, 2 washes were performed with PBS 1X and resuspended in 1 mL of medium. For 2D assays, cells were grown in 6-well plates at a concentration of 1 × 10^6^ and maintained for 24 h for attaching to the plate. For 3D assays, the hanging drop technique was employed. On the inside of a Petri dish lid, cells were placed in droplets at a concentration of 2 × 10^3^ cells/20 μL. The lid was gently turned upside down to prevent the droplets from falling. Cells were cultured for 2 weeks until spheroid formation. The change of culture medium was performed every 3 days. Cell cultures were treated with PTX (100 μM), NCTD (80 μM), and the combination of both drugs, for 6, 24, and 48 h. In addition, Rapamycin (RAPA, 10 μM) and Tunicamycin (TM, 7 μM) were used as controls for autophagy and endoplasmic reticulum stress, respectively.

### 4.2. Western Blot Analysis

Cells from 2D and 3D cultures were lysed in RIPA buffer (cat# 89900, Thermo Scientific^TM^, Waltham, MA, USA) with protease inhibitor (cat# P8340, Sigma Aldrich, St. Louis, MI, USA). Protein concentration was quantified with the BCA kit (cat# 23225, Thermo Scientific^TM^). Samples were adjusted to 30 μg/well and separated on a 12% SDS-PAGE gel. Electrophoresis was performed at 100 V for 1 h followed by a transference to a nitrocellulose membrane (BioRAD, Berkeley, CA, USA). The nitrocellulose membrane was blocked with 5% BSA (bovine serum albumin, cat# 30063-721, Gibco) in 0.5% TBS-Tween 0.5% (Tris-HCl 20 mM, pH 7.6, NaCl 150 mM, Tween 20 0.5%). Subsequently, anti-p62 (1:1000, cat# 39749, Cell Signaling Technology, Danvers, MA, USA) primary antibody was added and incubated overnight under constant agitation. Subsequently, 5 washes were performed with 0.5% TBS-Tween and incubated for 1 h with anti-rabbit antibody, HRP-linked (1:1000, cat# 7074, Cell Signaling Technology) and HRP conjugated anti-β-actin (1:3000, cat# 5125, Cell Signaling Technology). Finally, 5 washes were performed with TBS-Tween and SignalFire™ ECL Reagent (cat# 6883s, Cell Signaling Technology) was added for development on ChemiDoc^TM^ Touch (BioRad, Berkeley, CA, USA). The images obtained were analyzed with Image Lab software version 6.2 (BioRad).

### 4.3. Slices of the 3D Cultures of the Spheroids

Three-dimensional cultures in spheroids were collected in microtubes with the aid of a pipette. They were washed 2 times with PBS 1X and fixed in 100 μL of 4% paraformaldehyde for 24 h. Then, they were washed 2 times with PBS and placed in cryomolds. Tissue-Tek^®^ was added until completely covered. Subsequently, they were frozen at −20 °C and cut at 5 μm in the cryostat (HM525 NX, Epredia™ Kalamazoo, MI, USA). The slices were placed on 1% gelatin-coated slides. The slides were stored at −20 °C until use.

### 4.4. Immunofluorescence of 2D and 3D Cultures

For 2D staining, 5 × 10^5^ cells were cultured on sterile coverslips in a 6-well plate. Cells were cultured for 24 h at 37 °C in a humidified atmosphere with 5% CO_2_ to allow adherence. Drug treatments and controls were added as above. Slides with spheroid sections were thawed at room temperature. The same staining procedure was followed for both conditions. They were fixed with 4% paraformaldehyde for 5 min. Then, 2 washes were performed with PBS 1X and 0.25% NH_4_Cl was added for 5 min to quench endogenous fluorescence. Two washes were performed with 0.2% PBS-Triton for 5 min. Subsequently, blocking solution (PBS-Triton 0.2%, albumin 1%) was added for 1 h. The first anti-p62 antibody (1:200, cat# 39749, Cell Signaling Technology) diluted in the blocking solution was incubated for 2 h. Three washes were performed with 0.2% PBS-Triton. Mouse anti-rabbit FITC secondary antibody (1:200, sc-2359, Santa Cruz, Santa Cruz, CA, USA) diluted in blocking solution was kept for 1 h in the dark. Two washes were performed with 0.2% PBS-Triton. Slides were mounted in Fluoromount-GTM, with DAPI (cat# 4959-52, eBioscience, San Diego, CA, USA), and stored at 4 °C in the dark. Slides were observed under the LMS 5 Exciter confocal microscope (Zeiss, Oberkochen, Germany).

### 4.5. Statistical Analysis

Western blot assays were performed in triplicate. The relative expression was normalized based on β-actin expression. A Shapiro–Wilk test was performed to test the normality of the data. A one-way ANOVA test with a post hoc Dunnet test was performed for comparison analysis between treatments. The α value assigned for this study was <0.05. Statistical significance was considered when the *p* value was <0.05.

## Data Availability

The data presented in this work are available on request from the corresponding author.
